# Clinical trial landscape of diabetic retinopathy: global advancements and future directions

**DOI:** 10.1097/JS9.0000000000003475

**Published:** 2025-09-09

**Authors:** Haixing Cao, Yujie Zhang, Naiwen Zhang, Xiang Ma

**Affiliations:** Department of Ophthalmology, The First Affiliated Hospital of Dalian Medical University Dalian City, Liaoning Province, People’s Republic of China

Diabetic retinopathy (DR), a severe microvascular complication of diabetes, results from pathological processes triggered by chronic hyperglycemia, which induces multiple metabolic disturbances. In a sustained high-glucose environment, molecular events, such as elevated mitochondrial oxidative stress, accumulation of advanced glycation end-products (AGEs), and aberrant activation of the polyol pathway, culminate in pericyte apoptosis and disruption of the blood–retinal barrier[1]. According to the latest data from the International Diabetes Federation, the global diabetic population is projected to increase from 530 million (2021) to 780 million by 2045. Approximately 35% of these individuals may develop DR, potentially resulting in nearly 270 million cases of vision loss[2]. As a leading preventable cause of blindness in working-age adults, DR continues to pose a significant global public health burden due to rising rates of visual impairment and disability in the absence of timely intervention.

In clinical settings, anti-vascular endothelial growth factor (VEGF) drugs (such as ranibizumab and aflibercept) remain the first-line therapy for DR-related complications such as diabetic macular edema (DME). However, their efficacy is limited by the need for chronic intravitreal injections, variable patient response, emerging drug resistance, and inconsistent therapeutic outcomes[3]. Novel therapeutic approaches are increasingly under investigation in clinical trials. Corticosteroids (such as triamcinolone) are second-line options that reduce inflammation but can induce glaucoma and have short-lived effects, requiring intraocular pressure monitoring. Agents that improve microcirculation (such as calcium hydroxysulfonate) or act as antioxidants (such as epalrestat) can delay the progression of early-stage lesions. However, their ability to repair advanced structural damage is limited and frequently necessitates a combination with laser or surgical therapies. Traditional Chinese Medicine (such as Qi Ming Granules) can support metabolic regulation, although its effects are gradual and require further evidence-based validation[4]. Despite increasing global investment in DR drug development, approvals by major regulatory agencies (such as the U.S. Food and Drug Administration/European Medicines Agency/China’s National Medical Products Administration) remain sparse. Trials involving novel mechanisms frequently fail due to poor trial design, inappropriate endpoints, or insufficient recruitment, hindering translational progress and delaying paradigm shifts in DR management. Consequently, a systematic analysis of recent clinical trial progress and the underlying factors influencing success or failure holds significant practical and strategic importance.

The Informa Pharma Intelligence (INFORMA) database (pharma.id.informa.com) was systematically queried using the filters “Therapeutic Area: Ophthalmology” and “Disease Type: Diabetic Retinopathy” to identify all interventional trials on DR or DME registered between 1 January 2007 and 31 December 2024, regardless of phase (I–IV) or recruitment status (completed, active, terminated). Observational or retrospective studies, non-interventional records, trials involving non-DR retinal conditions or non-diabetic populations, duplicates, and studies lacking verifiable drug/intervention information were excluded. Two reviewers (H.C. and Y.Z.) independently extracted prespecified variables – trial phase, status, geographic location, primary endpoints, and drug characteristics (mechanism, target, class, and monotherapy vs. combination). Discrepancies were resolved by a third reviewer (C.M.) and verified against ClinicalTrials.gov and the European Union Clinical Trials Register. Risk of bias in randomized trials was assessed using the Cochrane RoB 2.0 tool, emphasizing adequacy of randomization, endpoint standardization [such as consistent Diabetic Retinopathy Severity Scale(DRSS)/Best-Corrected Visual Acuity (BCVA) metrics], and attrition bias (dropout ≥20% classified as high risk). Overall, 6852 records were screened, 4418 were excluded, and 2434 trials met the inclusion criteria. Of these, 78.3% were rated as low/moderate risk of bias, while 21.7% were high risk due to attrition or endpoint heterogeneity.

Our analysis highlighted significant trends in DR clinical development, including temporal distribution and status by trial phase, developmental progress of leading investigational agents, mechanisms of action, treatment modalities, and patterns in primary endpoint selection. Despite the extensive scope of the INFORMA database, certain limitations remain. These include possible selection bias inherent to the data source, lack of region-specific stratified analysis, and the exclusion of smaller or unpublished studies, which may limit comprehensiveness. In accordance with the TITAN 2025 guideline for transparent reporting of AI use in biomedical research, we provide a detailed checklist documenting every generative-AI contribution to this study[5].

Between 2007 and 2024, 2434 clinical trials for DR treatments were completed or initiated across 83 countries, demonstrating a steady annual increase. In 2024 alone, 53 Phase I, 83 Phase II, 90 Phase III, and 154 Phase IV trials were recorded, each representing the highest count. A significant increase began in 2021, when 114 Phase IV trials were conducted (Fig. 1A). This surge likely reflects stricter regulatory mandates for postmarketing studies to confirm clinical efficacy and long-term safety, alongside a growing emphasis on real-world evidence for reassessing clinical benefit and cost-effectiveness[6].

**Figure 1.**
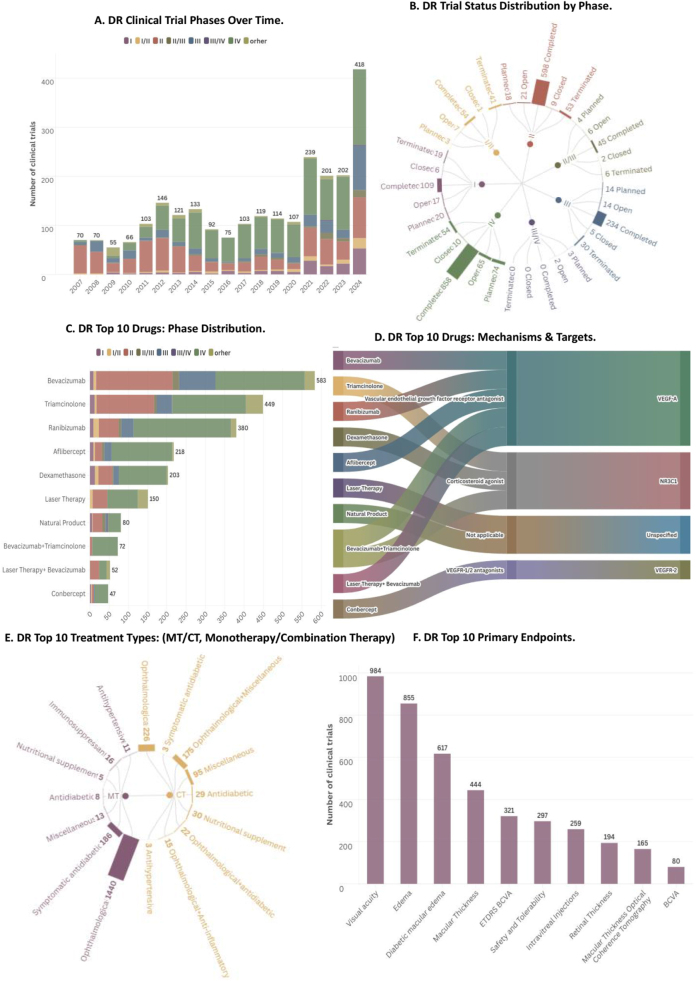
Clinical trial landscape of diabetic retinopathy in the world. (A) DR clinical trial phases over time; (B) DR trial status distribution by phase; (C) DR top 10 drugs: phase distribution; (D) DR top 10 drugs: mechanisms and targets; (E) DR top 10 treatment types: MT/CT, monotherapy/combination therapy; (F) DR top 10 primary endpoints.

Among all phases, Phase II and IV trials comprised 49.2% (598) and 70.9% (858), respectively. Phase I accounted for 109 trials (9%), and Phase III for 234 (19.4%). Termination rates were highest in Phase IV (54 trials, 4.5%), followed by Phase II (53, 4.4%), and Phase I/II (41, 3.4%). Ongoing Phase IV trials totaled 65 (5.4%), with 74 in the planning stage (6.1%), while other phases accounted for smaller proportions (Fig. 1B).

Across all trials, the top 10 drugs included eight monotherapies and two combination therapies, with anti-VEGF treatments dominating. The three most researched drugs were bevacizumab (583 trials, 24.0%), triamcinolone (449 trials, 18.4%), and ranibizumab (380 trials, 15.6%). In Phase IV, bevacizumab (231 trials, 20.4%) and ranibizumab (253 trials, 22.3%) led, while Phase II featured 199 bevacizumab trials (17.6%) and 51 ranibizumab trials (4.5%). Triamcinolone was the focus of 149 Phase II (13.2%) and 191 Phase IV trials (16.8%). Aflibercept appeared in 158 trials (13.9%) and dexamethasone in 125 (11.0%). Laser therapy was primarily studied in Phase IV (79 trials, 7.0%). Bevacizumab + triamcinolone combination therapy featured in 66 Phase IV trials (5.8%), while conbercept was investigated in 35 trials (3.1%) (Fig. 1C).

In total, bevacizumab (VEGF-A) and triamcinolone (Nuclear Receptor Subfamily 3 Group C Member 1 (NR3C1)] emerged as the most frequently studied drugs, followed by ranibizumab (VEGF-A) and aflibercept (VEGF-A). Dexamethasone (NR3C1) and conbercept (VEGF receptor-2) were evaluated in fewer trials. Additional therapies included laser therapy (158 trials) and natural products (80 trials). Combination approaches such as bevacizumab + triamcinolone (72 trials) and laser therapy + bevacizumab (56 trials) were also commonly explored (Fig. 1D).

Among the top 10 primary drugs tested, some ophthalmic drugs were combined with adjunct treatments (such as anti-diabetic drugs, immunosuppressants, antihypertensives, and nutritional supplements) for DR treatment. Ophthalmic monotherapy remained the predominant approach, accounting for 59.2% (1,440 trials), while ophthalmic drugs were used in 9.3% (226 trials) of combination therapies (Fig. 1E). In clinical studies, core efficacy endpoints include visual acuity and macular edema/thickness (Fig. 1F). Visual acuity is commonly assessed using Early Treatment Diabetic Retinopathy Study (ETDRS) BCVA or BCVA scales; macular or retinal thickness reflects retinal structural changes in the macular region, although definitions may vary across studies. Optical coherence tomography (OCT) is widely employed for precise measurement of macular thickness. DME refers specifically to DME, whereas “edema” is a more general term. Safety and tolerability metrics evaluate overall treatment safety, whereas intravitreal injection-related measures focus on procedural tolerance and patient experience.

Current DR treatment strategies, endorsed by the American Society of Retina Specialists (ASRS) and UK Consensus Working Group, emphasize a framework centered on anti-VEGF drugs (such as ranibizumab and aflibercept)^[7,8]^. For patients with DME, these agents are established as first-line therapy. For proliferative diabetic retinopathy (PDR) and severe non-proliferative diabetic retinopathy (NPDR), pan-retinal photocoagulation (PRP) remains a cornerstone of treatment. Anti-VEGF drugs can be used alone or in combination with PRP, particularly in patients capable of regular follow-up. Corticosteroids (such as triamcinolone) serve as second-line options, primarily when anti-VEGF therapies are contraindicated or ineffective. UK guidelines recommend corticosteroids for specific populations, such as pregnant patients. In advanced stages such as PDR, surgical interventions, including vitrectomy and laser photocoagulation, remain essential[9]. Vitrectomy is commonly preferred to manage complications such as vitreous hemorrhage and retinal detachment. Laser photocoagulation is critical for preventing further vision loss. Although the widespread use of anti-VEGF therapies has reduced reliance on surgical interventions in early-stage disease, surgical treatment remains indispensable for managing complications. This shift underscores the necessity to integrate pharmacologic and surgical strategies, highlighting the evolving and increasingly tailored approaches to DR management. While the ASRS and UK Consensus Working Group recognize the crucial role of anti-VEGF therapy, the ASRS emphasizes individualized treatment and follow-up management in PDR. Conversely, UK guidelines offer more detailed protocols for DME and special populations. A major clinical challenge is that therapeutic efficacy varies, although combinations of anti-VEGF agents, corticosteroids, and laser therapy are actively investigated in trials. Moreover, poor adherence to follow-up regimens can compromise anatomical and visual outcomes, indicating the need for optimized treatment strategies and improved patient management.

Conversely, clinical practice in China, shaped by variations in healthcare resource allocation, emphasizes the widespread use of anti-VEGF agents. This approach reflects a pragmatic balance between treatment cost and follow-up across diverse healthcare systems, and aligns with patterns observed in clinical trial distribution[10]. Future clinical trials should prioritize optimized study designs, incorporating standardized efficacy endpoints, such as the DRSS, and refined patient stratification. Moreover, new drug development must address mechanisms of resistance and low-responder populations, while leveraging new technologies, including AI and OCT angiography (OCTA), to improve screening and optimize patient selection.

Despite advances in DR treatment, significant challenges remain. Future management should shift toward more personalized and precise approaches. With ongoing progress in precision medicine, emerging strategies, such as neuroprotective agents and antioxidants, may offer breakthroughs. Furthermore, AI-assisted diagnostics, OCTA, and wide-angle imaging could enhance early detection and treatment efficiency. Ultimately, the focus will be on multidisciplinary, collaborative care, supported by large-scale, multicenter clinical data to refine treatment protocols and improve visual outcomes and overall quality of life for patients.

## Data Availability

The data supporting the findings of this study were obtained from the Informa Pharma Intelligence database (https://pharma.id.informa.com/), which is a commercially available source. The datasets analyzed during the current study are not publicly available due to licensing restrictions but may be accessed through a subscription to the Informa Pharma Intelligence platform. For further inquiries about data availability, please contact the corresponding author.
